# Phenolic Composition of Hydrophilic Extract of Manna from Sicilian *Fraxinus angustifolia* Vahl and its Reducing, Antioxidant and Anti-Inflammatory Activity in Vitro

**DOI:** 10.3390/antiox8100494

**Published:** 2019-10-18

**Authors:** Alessandro Attanzio, Antonella D’Anneo, Francesco Pappalardo, Francesco Paolo Bonina, Maria Antonia Livrea, Mario Allegra, Luisa Tesoriere

**Affiliations:** 1 Department of Biological, Chemical and Pharmaceutical Sciences and Technologies, Università di Palermo, 90123 Palermo, Via Archirafi, Italy; Alessandro.attanzio@unipa.it (A.A.); maria.livrea@unipa.it (M.A.L.); luisa.tesoriere@unipa.it (L.T.); 2 Department Analytics of R&D, Bionap s.r.l., C.da Fureria, Zona Industriale Ovest, Piano Tavola, 95032 Belpasso (CT), Italy; francesco.pappalardo@bionap.com; 3 Department of Pharmaceutical Science, Faculty of Pharmacy, Università di Catania, Viale Andrea Doria, 95125 Catania, Italy; boninaf@unict.it

**Keywords:** manna, *Fraxinus*, manna bioactivity, phytochemicals, antioxidant, reducing power, red blood cell oxidation, intestinal bowel disease model

## Abstract

Manna, a very singular vegetable product derived from the spontaneous solidification of the sap of some *Fraxinus* species, has long been known for its mild laxative and emollient properties. In this work, a hydro-alcoholic extract of manna (HME) from Sicilian *Fraxinus angustifolia* Vahl was investigated using HPLC-DAD to find phenol components and using chemical and biological in vitro assays to determine its reducing, antioxidant and anti-inflammatory capacity. We identified elenolic acid, tyrosol, hydroxytyrosol, catechin, fraxetin, verbascoside, gallic acid, procyanidin-B1, and luteolin 3,7 glucoside, in order of abundance. Measurements of total antioxidant activity by Folin-Ciocalteu reaction and ferric reducing ability (FRAP), as well as of scavenger activity towards ABTS•^+^, DPPH•, and perferryl-myoglobin radicals, showed that the phytocomplex effectively reduced oxidants with different standard potentials. When compared with vitamin E, HME also behaved as an efficient chain-breaking antioxidant against lipoperoxyl radicals from methyl linoleate. In cellular models for oxidative stress, HME counteracted membrane lipid oxidation of human erythrocytes stimulated by *tert*-butyl hydroperoxide and prevented the generation of reactive oxygen species, as well as the GSH decay in IL-1β–activated intestinal normal-like cells. Moreover, in this in vitro intestinal bowel disease model, HME reduced the release of the pro-inflammatory cytokines IL-6 and IL-8. These findings may suggest that manna acts as an antioxidant and anti-inflammatory natural product in humans, beyond its well-known effects against constipation.

## 1. Introduction

Known for centuries within the ethno-botanical field, the Sicilian “manna” is a very singular vegetable product obtained by the spontaneous solidification of the phloem sap collected from some *Fraxinus* species (*Oleaceae*). The production of manna requires a number of special conditions. High temperatures, low humidity and a low temperature range are necessary for the manna trees to be grown [1]. Some areas of the Madonie Mountains in Northern Sicily are ideal pedoclimatic niches for the cultivation of the ash trees, from which the sap is obtained and gathered in accordance with a traditional procedure. During the summer months, the tree barks are engraved by hand with the aid of specific tools to allow the sap to pour out. The spilled drops are allowed to solidify slowly and spontaneously along nylon threads, thus forming characteristic rod-shaped forms of whitish colour, known as *cannolo* manna [2,3]. At the same time, however, considerably more sap pours out along, adheres to the bark and thickens on the trunk from which it is recovered as a dirty and poor-quality product. Harvesting of the *cannolo* manna through the traditional method is tiring and time-consuming, with a very modest yield. This has recently led us to develop and patent a procedure for purifying the remarkably higher amounts of easily obtainable dirty manna (patent n.102015000061706).

Manna has long been acknowledged and used as a natural health remedy in popular medicine. Besides serving as a mild laxative, it is applied in dermatological preparations for its soothing and anti-aging effects, as well as in syrups, as fluidizing, expectorant and cough suppressant. Its consumption is also recommended to regulate intestinal and hepatic function [4]. Sugars, mono- and oligo- saccharides make up more than 80% of the dry weight of the *Fraxinus* manna [5,6]. Mannitol is the main constituent, representing 50% of the total sugars. In addition, a recent phytochemical characterization has revealed fatty acids and phenol components [5].

Measuring the reducing and antioxidant capacity of plant extracts is a way to determine their bioactivity, and illustraties the plant’s potential benefits to people in a healthy state and in patho-physiological conditions as well. In this study, we use a hydro-alcoholic solution to extract and identify by HPLC-DAD the phenolic components of manna from *F. angustifolia* Vahl and investigate for the first time reducing and antioxidant properties of the manna utilizing a number of well-established chemical and biological screening methods in solution and in cells. Moreover, anti-inflammatory effects of the extract were investigated in an in vitro model of intestinal bowel disease. Our results suggest that, thanks to its biophenols, manna may contribute to counteracting oxidative stress, maintaining cellular redox homeostasis and exerting anti-inflammatory effects.

## 2. Materials and Methods

### 2.1. Chemicals

Hydroxytyrosol, tyrosol, gallic acid, fraxetin, oleuropein, verbascoside, catechin, luteolin 3,7 glucoside, procyanidin B1, quercitin 3-O-glucoside, and elenoic acid were obtained from PhytoLab GmbH & Co. (Vestenbergsgreuth Germany). Fraxetin was from Sigma Chemical Co (St Louis, MO). Fetal bovine serum (FBS) was from Hyclone Perbio Sciences (Helsingborg, Skane Lan, Sweden). Human interleukin (IL)-1β, IL-6 and IL-8 enzyme-linked immunosorbent assay (ELISA) Kits II were from BD Biosciences Pharmingen (San Jose, CA, USA). HPLC-grade solvents and all other reagents and chemicals were from Sigma Chemical Co (St. Louis, MO, USA).

### 2.2. Plant Material and Preparation of Hydrophilic Manna Extracts (HME)

*Fraxinus angustifolia,* Vahl subsp *angustifolia*, representing the majority of the species and the richest source of manna among the ashes grown in the Madonie mountains, area of Castelbuono (Palermo, Italy) [3], was the source of manna in August 2018. Five lots of dirty, low quality manna from five different ashes were scraped from the bark, collected together and purified from rough contaminations following the procedure of patent n.102015000061706.

For the extraction procedure, manna samples (10 g) were completely dissolved in 30 mL distilled water. Then, two volumes of methanol (MeOH) were added and extraction carried out at room temperature (25 ± 2 °C) and in darkness for 1 h. afterwards, the mixtures were allowed to stand 4 h at 4 °C before centrifugation (3000 g, 10 min) to remove the crystallized sugars. The supernatants were withdrawn, filtered in a Büchner funnel through a 0.45 μm filter (Millipore, Billerica, MA) and submitted to rotary evaporation at 35 °C. Water residual from the solution was then eliminated by cryodessication. Dried samples of HME were stored at −80 °C and used within two months. Before the use, dried HME was resuspended in suitable volumes of 5 mM phosphate buffer saline, (PBS), pH 7.4.

### 2.3. Determination of Phenolic Compounds by HPLC-DAD

Dried HME was resuspended in distilled water (0.5 g manna equivalent/mL) which was purified using a cartridge Bond elut C18 (500 mg 6 mL) Agilent column (Santa Clara, CA, USA) according to the manufacturer’s instructions. Phenols were eluted with methanol (15 mL) and the solvent was removed under vacuum. The residue was dissolved in 1:1 (*v/v*) methanol-water at a final concentration of 30 mg manna equivalent/mL for HPLC analysis.

HPLC-DAD analyses were carried out in duplicate and performed using an Agilent 1100 Infinity, equipped with a diode array detector (DAD) and with a 150 × 4.6 mm i.d., 2,7 µm Ascentis Express C 18 column. The mobile phases were H_2_O/H_3_PO_4_ (99:1, solvent A) and MeOH/ACN/H_3_PO_4_ (49,5:49,5:1, solvent B) the gradient used was: concentration the solvent A of 95% going to 77% (34 min), maintain 77% (3 min), 74% (60 min), 60% (85 min), 20% (90 min) and 0% (92 min), total time 105 min. The column temperature was maintained at 25 °C. The flow was 1 mL/min and the injection volume was 15 μL. The profiles chromatograms were recorded from 190 to 500 nm and monitored at 240, 280, 330 e 346 nm ± 2 nm. The compounds were quantified by a five-point regression curve on the basis of the standards obtained from commercial suppliers. The retention time and the peak area were measured using the ChemStation software (Agilent Technologies Italia, Milan, Italy).

### 2.4. Reducing Capacity Tests

#### 2.4.1. Folin-Ciocalteu Reaction

Folin-Ciocalteu reaction, based on the reduction of phosphotungstic–phosphomolybdic acid (Folin-Ciocalteu’s reagent) to blue reaction products in alkaline solution, was used to quantify the total antioxidant activity (TAA). Aliquots (100 µL) of properly diluted HME (extracts from 2–20 mg manna, analysed in duplicate, in a 100 µL final volume) were mixed 3 mL of 2% sodium carbonate followed by the addition of 100 µL Folin-Ciocalteu’s reagent diluted 1:1 with water. The mixture was allowed to stand for 60 min at room temperature, in darkness. Then the absorbance was measured spectrophotometrically at 765 nm against a blank using a Beckman DU 640 spectrophotometer (Beckman, Milan, Italy). Quantitation was performed by curves constructed with gallic acid (5–100 µg/mL) and results were expressed as mg of gallic acid equivalents (GAE) per g manna.

#### 2.4.2. Ferric Ion Reducing Antioxidant Power (FRAP) Assay

Ferric ion reducing capacity of manna extract was tested using FRAP assay [7], according to Attanzio et al. [8].

### 2.5. Radical Scavenging Assays

#### 2.5.1. ABTS^+^ Radical Scavenging Assay

The radical-scavenging capacity of HME was evaluated using the 2,2′-azino-bis(3-etilbenzotiazolin-6-sulfonic acid (ABTS) radical cation decolourization assay [9].

#### 2.5.2. DPPH Radical Scavenging Assay

2,2-Diphenyl-1-picrylhydrazyl (DPPH) free radical scavenging activity was measured according to Brand-Williams et al. [10].

#### 2.5.3. Hypervalent Iron Mb Species Reduction Assay

Oxidation of horse met-myoglobin (met-Mb) to perferryl-myoglobin radical (•Mb[Fe^IV^=O]) was carried out, at 37 °C, in a reaction mixture containing 100 µM heme-protein in 0.1 M sodium acetate buffer, pH 5.0, supplemented with 100 µM H_2_O_2_, either in the absence or in the presence of 10 µL of properly diluted HME. Spectrophotometric scans were monitored at 400–800 nm before and 5 min after the H_2_O_2_ addition [11]. The hypervalent iron-concentration was calculated from the absorbance at 556 nm, the point at which the two forms differ most (ε = 3.6 mM cm^−1^) [12]. Myoglobin spectral changes in the visible region were measured on a DU 640 spectrophotometer equipped with a temperature controller.

In all assays described, samples of HME were analysed in duplicate, at three different dilutions, within the linearity range of the assay. The ABTS•^+^, DPPH• and •Mb[Fe^IV^=O] radical scavenging capacities of HME were quantified in comparison with Trolox, the water-soluble analogue of vitamin E, and the results expressed as Trolox equivalents (TE) per g of manna. Trolox was prepared as ethanol solution and the final concentration of ethanol in the assay mixture did not exceed 0.1%.

#### 2.5.4. Peroxyl Radical-Scavenging Assay in Solution

Peroxidation of methyl linoleate was performed by incubating 300 mM linoleic acid methyl ester (LAME) and 0.5 mM 2,2′-azobis(2,4-dimethyl-valeronitrile) (AMVN), in a final methanol volume of 1.0 mL, in a water bath at 37 °C, under air. Portions of the mixture (10 µL) were taken at intervals and injected onto a Supelco Supelcosil^TM^ (Bellafonte, PA, USA) LC-18 column (250 × 4.6 mm i.d., 5 µm), equilibrated, and then eluted with methanol at a flow rate of 1.0 mL/min. Quantitation was by reference to a standard curve constructed with known amounts of linoleic acid hydroperoxide (13(S)-hydroperoxy-(9Z,11E)-octadecadienoic acid). Aliquots (10 μL) of properly diluted HME or vitamin E (5 µM) in methanol were added to the methanol solution of methyl linoleate and allowed to equilibrate at 37 °C for 60 s, before adding the azoinitiator.

### 2.6. Antioxidant Activity in Red Blood Cells (RBCs)

Blood samples were obtained from healthy individuals by venipuncture with informed consent. Ethylenediaminetetraacetic acid (EDTA) (1 mg/mL blood) was used as an anticoagulant. RBCs were sedimented at 1000 g for 10 min and washed three times with phosphate-buffered saline (PBS), pH 7.4. Supernatant and buffy coat were carefully removed by aspiration after each wash.

A 1% suspension of RBCs in PBS was incubated with 100 µM *tert*-butyl hydroperoxide for 4 hs either in the absence or in the presence of various amounts of HME or vitamin E, used as a positive control. After incubation, samples of the mixture were withdrawn and malondialdehyde (MDA), as an index of lipid peroxidation, measured by HPLC as described by Tesoriere et al. [13].

### 2.7. Caco2 Cell Culture

Caco-2 cell line, derived from a human colon adenocarcinoma (American Type Culture Collection, ATCC), was used between passages 10 and 30 and cultured at 37 °C in a humidified atmosphere of CO_2_/air (5/95, *v/v*) in Dulbecco’s MEM with Glutamax supplemented with 25 mM HEPES, 10% (*v/v*) heat-inactivated Fetal Bovine Serum (FBS), 1% penicillin (1 × 10^3^ U/mL)-streptomycin (10 mg/mL), and 1% (*v/v*) non-essential amino acids (NEAA). Differentiated cells, grown to confluence for 18–21 days in 12-well plates replacing the media every 3 days (cell density of 60 × 10^3^ cells/cm^2^), were used for the experiments. For the treatments only freshly prepared and filtered (0.2 µm) HME were used. Cytotoxicity of the manna extract on Caco-2 cells was excluded by pilot studies using the Trypan Blue exclusion method and the MTT assay.

#### 2.7.1. Intracellular Reactive Oxygen Species (ROS) and GSH 

Differentiated monolayers of Caco-2 were preincubated with or without HME, at the indicated concentrations, for 1 h, and then were exposed to 25 ng/mL IL-1β for 24 h. Control cells were incubated with medium alone. After treatment, intracellular levels of ROS and GSH were cytofluorimetrically measured using the fluorescent probes 2′,7′-dichlorofluorescin diacetate (DCFDA) or 5-chloromethylfluorescein diacetate (CMFDA), respectively. Briefly, DCFDA or CMFDA (10 µM and 1 µM final concentration, respectively) were added to the medium 30 min before ending the treatment of the cells. The medium was then removed, and the cells were washed with PBS, resuspended in the same buffer and immediately analysed using flow cytometry (Epics XL^TM^, Beckman Coulter, Fullerton, CA) measuring fluorescence intensity in the FL-1 fluorescence channel at an excitation wavelength of 488 nm and emission wavelength of 530 nm. At least 10,000 events per sample were evaluated.

#### 2.7.2. Cytokines

IL-8 and IL-6 concentrations were quantified using ELISA kits according to the manufacturer’s instructions (DuoSet ELISA, R&D systems, Minneapolis, MN, USA).

### 2.8. Statistical Analysis

Results are given as means and standard deviations. Unless stated otherwise, three independent observations were performed for each experiment thrice replicated. Calculations and graphs were obtained using the INSTAT-3 statistical software (GraphPad Software, Inc., San Diego, USA) with a test for normality followed by ANOVA, with Tukey’s correction for multiple comparisons. In all cases, significance was accepted if the null hypothesis was rejected at the *p* < 0.05 level.

## 3. Results and Discussion

### 3.1. Polyphenols in HME 

**Figure 1:**
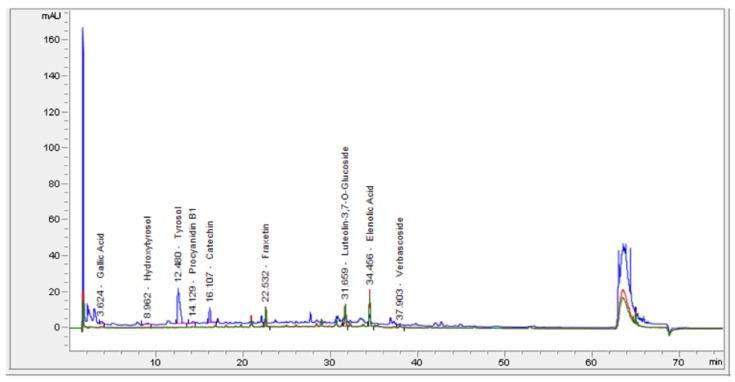
HPLC-DAD chromatogram of hydro-alcoholic extract of manna (HME) with identification of phenolic compounds. Revelation was at 280 nm, 330 nm and 346nm.

The quali/quantitative phytochemical content of vegetable extracts can vary by the extraction solvent used, which in turn will affect the eventual reducing/antioxidant activity of the extracts. Methanol has been reported to be the best extraction solvent for the large majority of plant phytochemicals [14,15]. We carried out a hydro-methanolic extraction of manna, followed by High-Performance Liquid Chromatography with Diode-Array Detection (HPLC-DAD) analysis, and identified polyphenol compounds by the absorption spectra and retention times of authentic compounds under our conditions (Figure 1). Elenolic acid and tirosol appear the most represented polyphenolic components, followed by hydroxytirosol (Table 1). Highly-polar polyphenol verbascoside, flavonoids such as cathechin, and B-type procyanidins, were also revealed, as well as gallic acid and the coumarin fraxetin (Table 1). Finally, a minor amount of luteolin 3,7 glucoside was also found.

In accordance with the chemotaxonomic closeness between *Fraxinus* and *Olea* genera, most of the identified compounds occur in the olive fruit and leaves, and in olive oil [16,17]. Nonetheless, we failed to find oleuropein, a major component of the genus *Olea*, also reported in *F. angustifolia* [18]. The high amount of elenolic acid, hydroxytirosol and tyrosol (Table 1) is possibly the result of hydrolysis of this seco-iridoid in its constituents, due to various factors, including light, temperature and bacterial enzymes [4]. Because of their demonstrated pharmacological potential, interest in biological and therapeutic effects of these phenols is huge [19].

Quercitin glucoside (quercitin-3-O-a-L-rhamnopyranosyl-(1->6)-b-D-glucopyranoside,) has been reported in the leaves and bark of *Fraxinus angustifolia (Hosny Phytochemistry 1998)*, however we did not find this compound in our manna extracts. 

In accordance with our findings, a recent phytochemical profiling of ethyl acetate extracts of manna from *Fraxinus excelsior* revealed the presence of simple phenolics, coumarins and iridoids, with elenolic acid and tyrosol and hydroxytyrosol as the most represented [5].

### 3.2. Reducing Capacity of HME

The Folin-Ciocalteu assay, based on the formation of blue-coloured products by the reaction between reducing compounds and reagent, is universally applied to reveal the total antioxidant activity (TAA) measured as phenols, and quantified as gallic acid equivalents, in various natural matrices, including dietary products [20]. HME showed a value of 1.22 ± 0.11 mg GAE per g of manna equivalent (Table 2).

The FRAP assay is based on the reduction of a Fe^III^-complex to the intensely blue coloured Fe^II^- form in the presence of reductants. The reducing activity of ascorbic acid is used as a reference. The HME showed a ferric ion reducing capacity of 0.22 ± 0.01mg AAE per g of manna equivalent (Table 2).

### 3.3. Radical Scavenging Capacity of HME 

The antiradical potential of the phenolic phytocomplex from HME was evaluated by the ability to scavenge ABTS●^+^ and DPPH•, or reduce the perferryl-Mb radical (•Mb[Fe^IV^=O]). The reducing activity was expressed as equivalents of Trolox; the water-soluble analogue of vitamin E was used as the reference compound.

The ABTS^+^ decolouration assay is widely considered appropriate to assess the antioxidant properties of plant foods [21]. Under our conditions, HME from 1 g of manna showed a quenching ability for the intensely coloured ABTS^+^ twenty times higher than Trolox (Table 2).

DPPH^●^ is a stable, deep purple radical whose reaction with an electron donor reducing agents [22,23] leads to loss of colour. It is also considered useful in providing information on the mechanism between the free radical and the antioxidant. We observed that HME, at 1g manna equivalent, showed a DPPH• radical quenching capacity comparable to that of 0.51 ± 0.04 µmol Trolox.

Perferryl-Mb, generated by reaction of Met-Mb with hydrogen peroxide [24], is a very reactive two-electrons oxidized form of Met-Mb, comprising an oxoferryl moiety and a globin-based tyrosyl radical (●Mb[Fe^IV^=O]) [25]. The measurement of the deactivation rate of perferryl-Mb by plant phenols is a useful model to evaluate the reducing capacity of these compounds [26,27]. HME showed a perferryl-Mb reducing rate of 1.13 ± 0.09 mmol Trolox/g manna equivalent for minute (Table 2). The radical trapping capacity of compounds is not exclusively related to their redox potentials but depends also on their distribution in the phase of the oxidizing probe. It is known that interaction with the radical site in the Mb, or access to the heme hydrophobic pocket, requires suitable size, three-dimensional structure and physico-chemical feature of the reducing compound [28]. Our results may, therefore, indicate that phenolic components with such molecular properties are presents in the manna extract involved in the observed reducing activity. Flavonoids, including catechin, have been shown to be reductants of perferryl myoglobin radicals [28].

The capacity of manna components to interact and quench the Mb [Fe^IV^=O] is highly interesting, since such oxoferryl radical species are also formed during the catalytic cycle of eme-enzymes, such as cyclooxygenase [29] and myeloperoxidase [30], suggesting that manna may have the potential to intervene under inflammatory conditions.

On the whole, the results presented in Table 2 show that the mixture of polyphenols in manna can neutralize a number of different oxidant or radical species, in assays characterized by chromogenic redox reagents with different standard potentials. When considered altogether these assays provide a reliable idea of the protective potential of manna. From a biological perspective, according to data from the literature reporting results of these assays applied to fruits, vegetables and their derivatives [23,31,32], the TAA of manna appears comparable to that reported for honeydew and the most fruits consumed in the Mediterranean diet, such as grapes, peaches or nectarines [33]. In addition, taking into account the values reported for sweetener products, the ABTS radical reducing capacity of 1 g manna appears twice as high as that of one serving of maple syrup and raw cane sugar, and comparable to 20 mL of honey [34].

### 3.4. Antioxidant Activity of HME in Solution 

**Figure 2:**
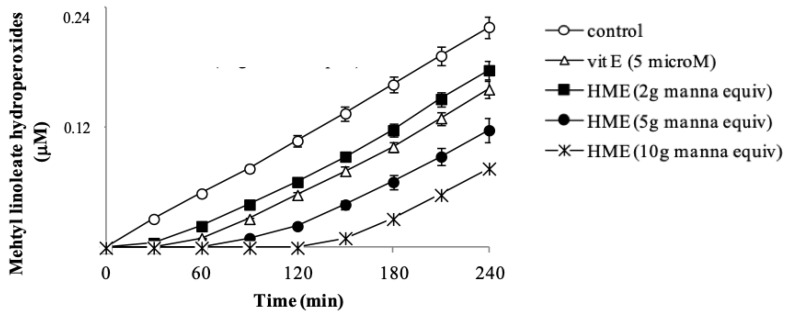
2,2′-azobis(2,4-dimethyl-valeronitrile) (AMVN)-induced oxidation of methyl linoleate in methanol in the absence (control) or in the presence of HME. Oxidation conditions and HPLC analysis of methyl linoleate hydoperoxides are reported in methods. Vit E was used as a positive control. Each point represents the mean ± SD of three determinations carried out in triplicate with different incubation mixtures.

Due to the abundance of membrane phospholipids, lipoperoxyl radicals are the most easily generated radical species in cells under either endogenous or exogenous oxidative insult. Phenol and polyphenol compounds are potent reductants capable of neutralizing peroxyl radicals [35]. The potential of HME to inhibit lipid oxidation was checked in methyl linoleate methanol solutions oxidized by the azo-initiator AMVN. Vitamin E, the most important lipid antioxidant, was used as the positive control. In the absence of manna, the addition of AMVN started a lipid oxidation evident as a steady formation of lipid hydroperoxides, measured by HPLC (Figure 2). A net concentration-dependent delay of the hydroperoxide formation (lag time) was observed in the presence of HME (Figure 2), indicating that phenol components of manna had inhibited lipid oxidation. Vitamin E at 5 µM caused a lag time of methyl linoleate hydroperoxide formation of 30 min (Figure 2). Interestingly, under our conditions, phenol components in 1 mg manna show an antioxidant capacity two-times higher than 1 µM Vitamin E. 

In these assays, antioxidant and substrate actually compete for thermally generated peroxyl-radicals through decomposition of azo-compounds [36]. It may be important to mention that the profile of the inhibition curve in the presence of manna, showing a definite lag time (Figure 2), provides evidence that extract components actually react with and scavenge lipoperoxyl radicals [36]. Regarding this, it is known that hydroxytyrosol behaves as a chain-breaker by donating a hydrogen atom to lipoperoxyl-radicals [37].

### 3.5. Antioxidant Activity of HME in Human Cells

Human RBCs are particularly useful in the evaluation of the antioxidant properties of several compounds [38,39]. These cells are particularly susceptible to endogenous oxidative damage because of their specific role as oxygen carriers and for the abundance of polyunsatured lipid in their membranes. When RBCs are exposed in vitro to organic hydroperoxides like *tert*-butyl hydroperoxide, the initial decomposition of the peroxide in the presence of heme iron generates methemoglobin (met-Hb), H_2_O_2_ and a number of oxyradicals from the hydroperoxide [40] that attack unsaturated lipids in membranes, finally generating MDA.

**Figure 3:**
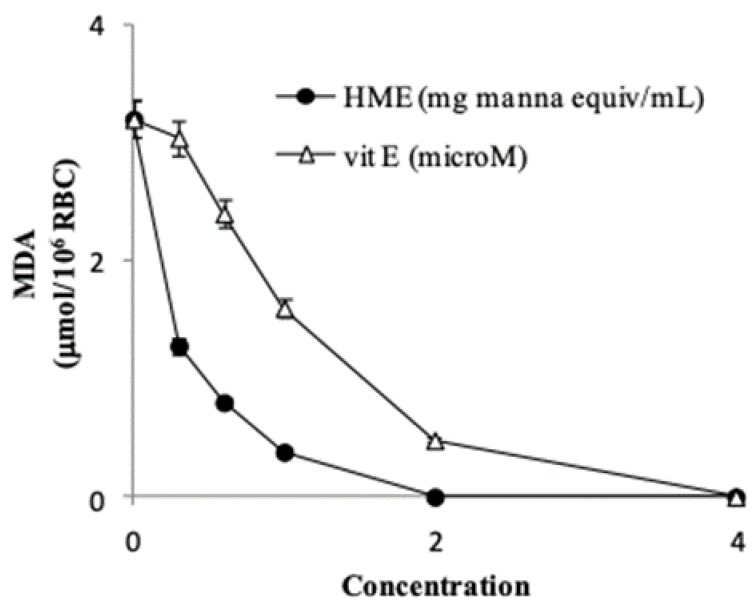
HME inhibits membrane lipid oxidation of human erythrocytes stimulated by *tert*-butyl hydroperoxide. Cell oxidation conditions and HPLC analysis of malondialdehyde (MDA) are reported in the methods section. Vit E was used as a positive control. Each point represents the mean ± SD of three determinations carried out in triplicate with different incubation mixtures.

Treatment of RBCs (1% HT) with 100 µM *tert*-butyl hydroperoxide caused the formation of 3.2 ± 0.29 µmol MDA/10^6^ cells (*n* = 4) after 4 h incubation. HME from 0.3 to 2.0 mg manna inhibited the MDA production in a dose-dependent manner (Figure 3), with a calculated IC_50_ (i.e., the amount that inhibits MDA formation by 50%) of 0.24 ± 0.04 mg manna equivalent (*n* = 4). 

In our system the IC_50_ of vitamin E, used as a positive control, was 1.1 ± 0.08 µM (*n* = 4) (Figure 3). Overall, these findings indicate that the phenol components of manna may act as very effective antioxidants and protect red blood cell membrane lipids from oxidation. It is worth mentioning that hydroxytyrosol at low micromolar concentration has been shown to protect red blood cells from oxidative damage stimulated by hydrogen peroxide [41].

### 3.6. Anti-Oxidant Effects of HME in Differentiated Caco-2 Cells Stimulated by IL-1β

ROS play important roles as signaling molecules in normal physiology, as well as in inflammatory diseases and cancer [42,43]. In this scenario, the effects of redox-active phytochemicals that may maintain the cell-specific redox tone was investigated. 

**Figure 4:**
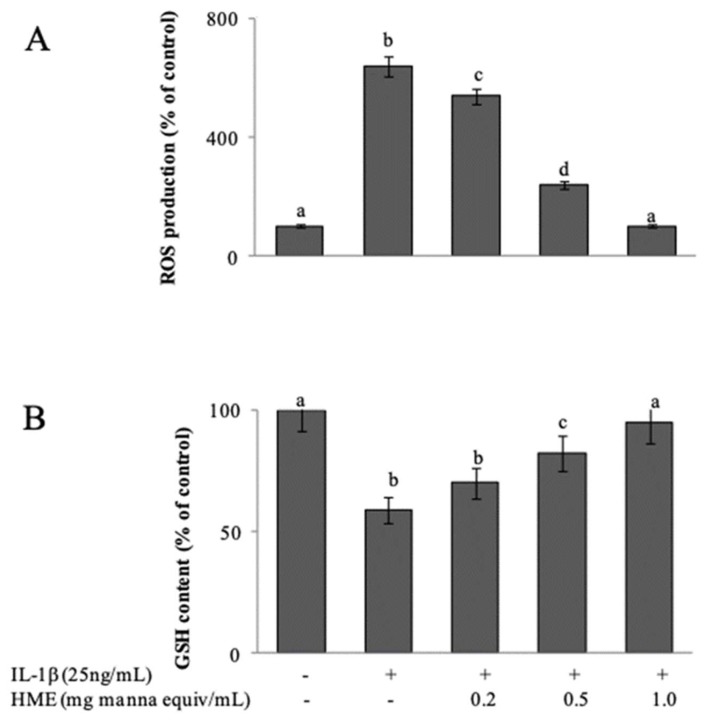
HME inhibits the generation of reactive oxygen species (**A**) and the GSH decay (**B**) in IL-1β-activated intestinal normal-like cells. Arithmetic means ± SD (*n* = 9) of2’,7’-dichlorofluorescin diacetate (DCFDA)-associated mean fluorescence intensity (MFI) (**A**) and 5-chloromethylfluorescein diacetate (CMFDA)-associated MFI after 24 h incubation of differentiated CaCo 2 cells with IL-1β preceded by 1 h pre-treatment in the presence of HME or vehicle. Bars with different letters are significantly different with *p* < 0.05 (Anova associated with Tukey test).

Differentiated Caco-2 cells exposed to IL-1β are considered an established model of an inflamed human intestinal barrier [44], in which stimulated ROS over-production triggers the activation of redox-sensitive intracellular signalling cascades that promote the inflammatory response. When 15 day-post confluent IL-1β-treated Caco-2 cell monolayers were treated for 3 h with IL-1β, cytofluorimetric analysis with DCFDA showed a net increment of intracellular ROS levels respect to untreated cells (Figure 4A). As a consequence, the intracellular GSH concentration, cytofluorimetrically measured by CMFDA probe, appeared significantly decreased of about 40% (Figure 4B). Co-incubation of the cells with IL-1β and HME in the range from 0.2 to 1.0 mg/mL manna equivalent, inhibited both ROS generation (Figure 4A) and the GSH loss (Figure 4B) in a concentration-dependent manner. HME at 1 mg manna equiv /mL was able to completely prevent the IL-1β-induced oxidative stress of the cells (Figure 4).

### 3.7. Anti-Inflammatory Effects of HME in Differentiated Caco-2 Cells Stimulated by IL-1β

**Figure 5:**
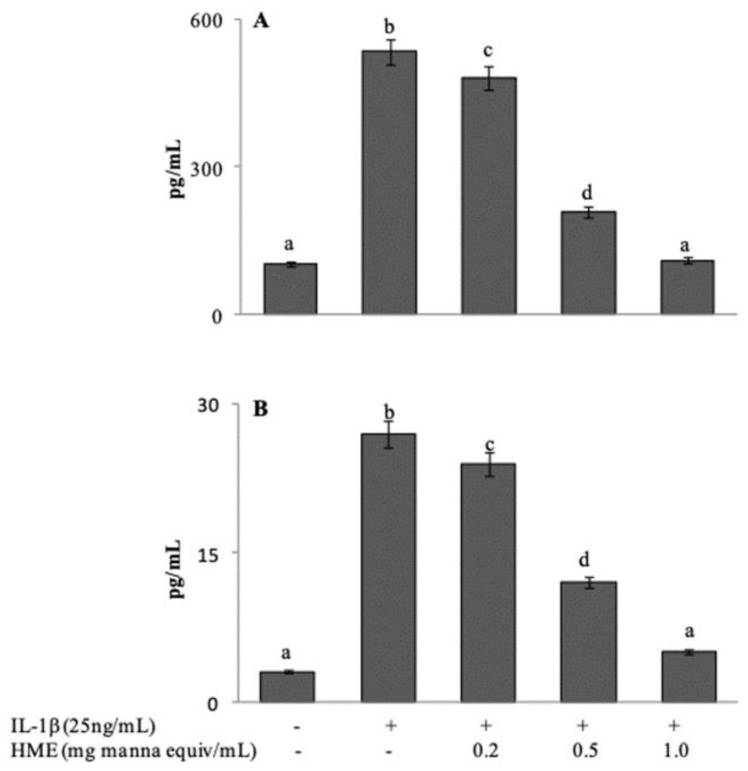
HME inhibits the release of the pro-inflammatory cytokines IL-8 (**A**) and IL-6 (**B**) in IL-1β-activated intestinal normal-like cells. Arithmetic means ± SD (*n* = 9) of the values after 24 h incubation of differentiated CaCo 2 cells with IL-1β preceded by 1 h pre-treatment in the presence of HME or vehicle. Release of cytokines in the cell incubation medium was measured by ELISA. Bars with different letters are significantly different with *p* < 0.05 (Anova associated with Tukey test).

Following exposure to IL-1β, Caco-2 cells express and release a number of inflammatory mediators. Release of IL-8 and IL-6 in the cell medium after IL-1β stimulation, either in the absence or in the presence of HME, was assessed in comparison with untreated Caco-2 cells. In our experimental system, basal levels were evaluated at 102.71 ± 18.42 and 3.26 ± 0.33 pg/mL for IL-8 and IL-6 respectively (Figure 5A,B). Stimulation with IL-1β at 25 ng/mL for 24 h induced a 5.2-fold increase of IL-8 secretion (534.09 ± 21.39 pg/mL), and an 8.5-fold increase (27.71 ± 3.01 pg/mL) of IL-6. Co-incubation of the cells with HME in the range between 0.2 and 1.0 mg/mL, concentration-dependently reduced the release of both cytokines with the system reaching control values at the highest HME concentration (Figure 5A,B). Our results suggest that the phytocomplex of manna can attenuate the inflammatory response of intestinal cells.

## 4. Conclusions

The medicinal properties of *Fraxinus* species have long been known and find application in folk remedies, as well as in contemporary medicine [18,45]. Studies on the crude extracts from bark, leaves and flowers have revealed significant antimicrobial, antioxidative, photodynamic damage prevention, wound healing, anti-inflammatory, immune-modulatory and antiviral activities [45]. This work reports, for the first time, evidence regarding the bioactive properties of the sap from some *Fraxinus angustifolia* Vahl, the so-called manna, which may provide a scientific basis to the long and traditionally reported health benefits of this product.

We here report the polyphenol composition of a hydro-alcholic extract of manna and demonstrate its reducing and antioxidant capacity, in water as well as in lipid phase. HME was effective in scavenging several reactive radicals including those derived from heme-proteins, showed lipoperoxyl radical-scavenging capacity in solution and, more importantly, provided antioxidant protection in complex environment as human red blood cells.

Furthermore, the manna extract showed anti-inflammatory effects in IL-1β treated Caco-2 cells, highly regarded as a model of intestinal bowel disease.

The quite high levels of phenols/polyphenols in our extracts, with hydroxytirosol, as one of the major components, can account for the observed reducing and free-radical scavenging properties of manna.

Known health benefits of manna have mostly been ascribed to, and are consistent with the properties of mannitol, an osmotically active cell-compatible polyol [46]. The antioxidative and anti-inflammatory effects here ascertained add potential functional characteristics to this plant product.

Notwithstanding the wide array of experimental approaches employed in this work, aiming to assess the antioxidative and anti-inflammatory potential of HME, a limitation of the present study is related to the in vitro approach. Along these lines, and taking into account the present results suggesting the potential for HME to control redox-regulated signaling pathways involved in cell homeostasis, studies on the activity of manna in both normal and pathological in vivo systems are in progress in our lab.

## Figures and Tables

**Table 1:** Phytochemicals identified in manna extract from Fraxinus angustifolia Vahl.

Class	Compound	mg/Kg
***Simple Phenols***	Gallic acid	4.12 ± 0.32
***Coumarins***	Tyrosol	36.66 ± 0.25
***Phenylpropanoids***	Hydroxytyrosol	13.33 ± 0.40
***Flavonoids***	Fraxetin	4.74 ± 1.02
***Secoiridoids***	Verbascoside	4.5 ± 0.21
***Seco-iridoid derivative***	Catechin	5.86 ± 0.52
	Luteolin 3,7 glucoside	1.45 ± 0.35
	Procyanidin B1	3.69 ± 0.34
	Quercitin 3-O-glucoside	n.d.
	Oleuropein	n.d.
	Elenolic acid	250 ± 4.78

Manna was extracted and treated as described in Methods. The values are the mean ± SD of two different extractions and three HPLC runs for each sample (*n* = 6). n.d., not detected.

**Table 2:** Reducing capacity and Radical scavenger activity of manna extract.

TAA ^a^ (mg GAE ^b^/g)	FRAP (mg AAE ^c^/g)	ABTS•^+^ (mmol TE ^d^/g)	DPPH• (mmol TE ^d^/g)	•Mb[Fe^IV^=O] Deactivation Rate (mmol TE ^d^/g min^−1^)
**1.22 ± 0.11**	**0.22 ± 0.01**	**23.4 ± 1.51**	**0.51 ± 0.04**	**1.13 ± 0.09**

^a^ TAA; Total Antioxidant Activity. ^b^ GAE; Gallic Acid Equivalent. ^c^ AAE; Acid Ascorbic Equivalent. ^d^ TE; Trolox Equivalent. Values are the mean ± SD of four determinations carried in triplicate.

## Patents

Purified manna in the present study was obtained following the procedure of patent n.102015000061706.
